# The role of dyadic combinations of infants' behaviors and caregivers' verbal and multimodal responses in predicting vocabulary outcomes

**DOI:** 10.1111/infa.12626

**Published:** 2024-09-25

**Authors:** Anika van der Klis, Caroline Junge, Frans Adriaans, René Kager

**Affiliations:** ^1^ Institute for Language Sciences Department of Languages Literature and Communication Utrecht University Utrecht The Netherlands; ^2^ Experimental Psychology Department of Developmental and Experimental Psychology Utrecht University Utrecht The Netherlands

## Abstract

There is robust evidence that infants' gestures and vocalisations and caregivers' contingent responses predict later child vocabulary. Recent studies suggest that dyadic combinations of infants' behaviors and caregivers' responses are more robust predictors of children's vocabularies than these behaviors separately. Previous studies have not yet systematically compared different types of dyadic combinations. This study aimed to compare the predictive value of (a) frequencies of infants' behaviors (vocalisations, points, and shows + gives) regardless of caregivers' responses, (b) frequencies of infants' behaviors that elicited verbal responses, (c) frequencies of infants' behaviors that elicited multimodal responses, and (d) frequencies of infants' behaviors that did not elicit any responses from caregivers. We examined 114 caregiver‐infant dyads at 9–11 months and children's concurrent and longitudinal vocabulary outcomes at 2–4 years. We found that infants' points elicited a large proportion of verbal responses from caregivers which were related to children's later receptive vocabularies. We also found that only shows + gives that elicited caregivers' responses related to infants' concurrent gesture repertoires. In contrast, infants' behaviors that did not elicit responses negatively related to child vocabulary. The results highlight the importance of examining dyadic combinations of infants' behaviors and caregivers' responses during interactions when examining relations to children's vocabulary development.

## 1 INTRODUCTION

Before the onset of their first words, infants start producing vocalisations and gestures to communicate. Gestures are predictors of children's language development (e.g., Brooks & Meltzoff, 2008; Rowe et al., 2008; Rowe & Goldin‐Meadow, 2009). This is most often researched for deictic gestures. Deictic gestures include points (index‐finger extensions), shows (holding out an object), and gives (passing on an object) which appear relatively early in children's development (e.g., Bates et al., 1979; Capone & McGregor, 2004; Frank et al., 2021). Children's points have been particularly well‐studied in a broad age range of children and strongly predict their concurrent and longitudinal language outcomes (for meta‐analyses, see Colonnesi et al., 2010; Kirk et al., 2022), although studies suggest that children's shows + gives (i.e., a combined category including both showing and giving gestures) may be precursors to points (Cameron‐Faulkner et al., 2015; Choi et al., 2021) and better predictors of children's later vocabulary skills than points when measured early from 10 to 12 months of age (Choi et al., 2021; Donnellan et al., 2019). The meanings of deictic gestures depend on the immediate context in which they are being used. Gestures and speech alike involve understanding the sign‐referent relationship. Early gestures could reflect children's general ability to learn symbols which paves the way to spoken language (Namy & Waxman, 1998). The link between early gestures and children's vocabulary development could therefore stem from the underlying understanding of the symbolic and referential use of gestures and words (see Werner & Kaplan, 1963). Therefore, infants' shows + gives and points could be related to their vocabulary outcomes through their shared reliance on symbolic and referential meaning.

Infants expect their caregivers to respond with gaze alternation and contingent comments (i.e., joint attention) to their shows + gives and points from at least 10 and 12 months of age respectively, which suggests that infants produce these gestures with the goal to share attention and interest with others (Boundy et al., 2019; Liszkowski et al., 2004). Crucially, language learning occurs in social contexts constructed by the infant and the caregiver (Renzi et al., 2017). Research shows that caregivers' immediate responses to infants' communicative behaviors are related to children's vocabulary development (e.g., Donnellan et al., 2019; McGillion et al., 2013; Olson & Masur, 2015; Wu & Gros‐Louis, 2014). It has been hypothesised that infants can immediately learn from the verbal contents of the response if the response is semantically and temporally contingent on the infants' behavior (see Tamis‐LeMonda et al., 2014). Mothers respond more often to infants' gestural than non‐gestural communicative behaviors (Olson & Masur, 2013; van der Klis et al., 2023). More specifically, infants' points have been found to elicit more verbal labeling responses from adults (Kishimoto et al., 2007; Wu & Gros‐Louis, 2015). For example, when the infant points at a doll, the caregiver may immediately respond with “That's a doll!”. This could make it easier for the infant to match the phonological form “doll” onto the object. Olson and Masur (2015) demonstrated that caregivers' verbal labeling responses to infants' gestures completely mediated the relationship between infants' gestures and their vocabulary outcomes. Since infants' points have been found to elicit more labeling responses from caregivers, this could be one of the mechanisms through which infants' points are a robust predictor of children's vocabulary outcomes. Likewise, studies do not typically find an effect of infants' vocalisations on children's vocabularies by themselves, while adults' semantically contingent responses directly elicited by infants' vocalisations – or dyadic combinations of infants' vocalisations and caregivers' contingent responses – can significantly predict children's vocabulary skills (Donnellan et al., 2019; Gros‐Louis et al., 2014; Lopez et al., 2020; McGillion et al., 2013). Yet, a recent study using all‐day recordings shows that infants also vocalise frequently while being alone, suggesting that infants might learn to produce speech by independently exploring their own vocal abilities (e.g., Long et al., 2022). The question remains whether this independent exploration relates to children's language development.

### 1.1 Multimodal language input

Previous studies have primarily focused on caregivers' verbal responses to infants' behaviors. However, there is ample evidence that caregivers' nonverbal cues can contribute to word learning. Nonverbal cues in caregivers' responses toward infants, such as handing over a toy, pointing, or smiling, predict children's vocabulary outcomes (Pearson et al., 2011; Ruddy & Bornstein, 1982). For example, when the infant points at a rattle, the caregiver may pick it up and shake the rattle while saying “What a nice rattle!”. This provides the infant with both a verbal and a visual cue as to what “rattle” refers to. Children can use gaze direction, body orientation, and index‐finger pointing as cues to map words onto objects (e.g., Baldwin et al., 1996; Grassmann & Tomasello, 2010; Kory Westlund et al., 2017; Verhagen et al., 2019). Such nonverbal cues reduce any referential ambiguity in the language input. In addition, Ger et al. (2018) found that the proportion of caregivers' responses to infants' points that was multimodal (verbal + nonverbal) at 10 months positively predicted infants' points at 12 months, suggesting that caregivers' multimodal responses can reinforce infants' points. Similarly, Cameron‐Faulkner et al. (2015) found a positive correlation between the frequency with which mothers acted upon target objects (e.g., by playing with it) after their infants produced shows + gives and infants' index‐finger pointing frequency at 12 months. During such interactions, infants choose the object of interest, and their caregiver generally commented and/or acted upon it (e.g., by accepting and manipulating it), and then returned the object to the infant (Cameron‐Faulkner et al., 2015). Lastly, a recent study suggests that caregivers' multimodal responses could increase the duration of children's looks at the toy (i.e., enhance attention) (Chen et al., 2021). These studies suggest that caregivers' multimodal responses could be useful in reinforcing infants' communicative behaviors during interactions, providing additional visual cues to reduce the referential ambiguity in the learning environment, and increasing infants' attention – subsequently facilitating children's vocabulary development.

Despite the robust evidence of the facilitative role of nonverbal behaviors, previous studies examining the link between caregivers' responses and children's vocabulary outcomes typically focused on caregivers' verbal responses (e.g., Donnellan et al., 2019; McGillion et al., 2013; Olson & Masur, 2015; Wu & Gros‐Louis, 2014). Recently, Choi et al. (2021) annotated caregivers' verbal and nonverbal responses to infants' shows + gives and points. They found that caregivers respond more often to infants' shows + gives than infants' points when the infants were 10 months of age, and only infants' shows + gives at 10 months could predict children's vocabulary outcomes at 18 months. The authors hypothesised that shows + gives at 10 months were a better predictor of children's vocabularies because they elicited more responses from caregivers than points at this early age. Although Choi et al. (2021) annotated caregivers' verbal and nonverbal responses to infants' shows + gives and points, they did not distinguish between these responses, and they did not examine how they differentially relate to children's vocabulary outcomes. In a previous study, we found that 9‐ to 11‐month‐old infants' gives elicited more multimodal (verbal + nonverbal) responses from caregivers, while infants' points elicited more verbal responses from caregivers (van der Klis et al., 2023). If infants' shows + gives are related to children's vocabulary outcomes due to the high rates of responses they elicit from caregivers at this early age, we would expect that caregivers' multimodal responses also contribute to the relationship between infants' shows + gives and children's vocabulary outcomes. To our knowledge, no prior studies have examined the effects of caregivers' multimodal responses on children's vocabulary outcomes.

### 1.2 Research aims

This study aimed to assess whether dyadic combinations of infants' vocalisations and gestures (shows + gives and points) and caregivers' verbal and multimodal responses during a free play session at 9–11 months of age improves the predictive value of these infants' behaviors for explaining variation in children's concurrent and long‐term vocabulary outcomes at 2–4 years of age. To examine this, we compared the predictive value of four subsets of individual and dyadic behaviors: (a) frequencies of infants' individual behaviors (vocalisations, points, and shows + gives) regardless of caregivers' responses, (b) frequencies of infants' behaviors combined with caregivers' verbal responses, (c) frequencies of infants' behaviors combined with caregivers' multimodal responses, and (d) frequencies of infants' behaviors that did not elicit any responses from caregivers. This allows us to pit different theories that aim to explain how infants' behaviors relate to their language outcomes against each other. On the one hand, if the relationship between infants' gestures and spoken language is driven by their common underlying understanding of referentiality (e.g., Werner & Kaplan, 1963) or through an independent exploration of their vocal and gestural abilities (e.g., Long et al., 2022), we would expect infants' behaviors to be predictors of children's language outcomes regardless of caregivers' responses. On the other hand, if infants' behaviors relate to their language development because infants create more word‐learning opportunities for themselves by eliciting informative responses from caregivers (e.g., Donnellan et al., 2019), we would expect dyadic behaviors – where infants' behaviors elicited contingent responses from caregivers – to more robustly predict children's language outcomes compared to infants' behaviors alone. Although the added value of caregivers' multimodal responses has not yet been studied, we expect that caregivers' multimodal responses additionally facilitate children's vocabulary development by reducing referential ambiguity in unclear or novel speech, by reinforcing infants' behaviors during interactions, and/or by increasing the infants' attention to toys (e.g., Baldwin et al., 1996; Cameron‐Faulkner et al., 2015; Yu et al., 2019; Ger et al., 2018; Grassmann & Tomasello, 2010). The results add to our understanding of the facilitative role of infants' prelinguistic vocalisations and gestures in children's vocabulary development.

## 2 METHODS

### 2.1 Participants

The data for this study are derived from YOUth, a longitudinal cohort study part of Utrecht University and University Medical Center Utrecht (Onland‐Moret et al., 2020). YOUth has repeated measurements at regular intervals (“waves”). The sample for this study consisted of 114 infants (65 females; 49 males) at 9–13 months of age (*M* = 10.7, *SD* = 0.9) during Wave 1 and their caregivers (90 mothers; 24 fathers). This is the same sample reported in van der Klis et al. (2023), but we excluded three participants for the current study: one participant because the child has developmental language disorder, one participant because they were multilingual, and one participant because the child suffered from many ear infections during development. For the present study, we analyzed these infants' concurrent and longitudinal vocabulary outcomes. When the children were followed up in Wave 2, they were 2–4 years old (*M* = 2.7, *SD* = 0.5). There were approximately one to 3 years (*M* = 1.8, *SD =* 0.5) in between measurement waves, randomly varying per participant. Most caregivers in the sample completed a college or university degree (83.5%). The YOUth cohort study is carried out in accordance with The Code of Ethics of the World Medical Association (Declaration of Helsinki), and all caregivers have signed informed consent for each child before any assessment or data collection. The study was approved by the medical ethical committee of the University Medical Center Utrecht (application number 14–7–221). Children received a picture book after participating in Wave 1 and a frog umbrella after participating in Wave 2.

### 2.2 Materials and procedure

#### 2.2.1 Wave 1: Caregiver‐infant interaction

During the lab visit for Wave 1 when the infants were 9–11 months of age, the infants and their caregivers were asked to sit on a blanket with a standard set of toys (a baby doll with a milk bottle, a green toy car, a Bumba pop‐up toy, a sun‐shaped rattle, a shape sorter, and a picture book) in a sparsely furnished room. Caregivers were instructed to play as if they were at home. Three Dome cameras that can be moved and zoomed in and out were placed around the blanket and one fixed camera filmed an overview of the scene. To capture sound, a fixed, standing (Sennheiser ME64/K6P condenser) microphone was positioned next to the blanket. After reading out the instructions, the research assistant would take place behind a screen, so they were completely out of view. The dyads completed five 3‐min sessions consecutively, resulting in a total of 15 minutes. The tasks were completed in a fixed order: free play, playing with a shape sorter, reading a picture book, again free play, and cleaning up. For the present study, we analyzed the two bouts of free play (6 minutes in total) per dyad.

#### 2.2.2 Wave 1: N_YOUth_‐CDI 1

After the lab visit, caregivers were instructed to fill out the N_YOUth_‐Communicative Development Inventory (CDI) 1, which is our adapted version of the N‐CDI 1 and N‐CDI‐WG (Zink & Lejaegere, 2003). We replaced or removed 12 typical Flemish words with synonyms that are more common in Standard Dutch spoken in the Netherlands (e.g., we removed *mantel* from *jas(je)/mantel* (“coat”)). We also included the list containing 65 gestures and actions from the full‐length N‐CDI‐WG (Zink & Lejaegere, 2002). This scale contains “early gestures” including the first communicative gestures (e.g., points, shows, and gives) and games and routines (e.g., playing peekaboo) and “late gestures” including actions with objects (e.g., eating with a spoon or fork) and pretending to be a caregiver (e.g., pretending to feed a doll). For infants in this age group, the gesture scale appears to be a relatively reliable measure of early vocabulary. Compared to the vocabulary scales for infants, the gesture scale does not suffer from floor effects for this age group, and it is the only scale that correlates with children's longitudinal vocabulary outcomes (van der Klis et al., 2024). For the 103 vocabulary items, caregivers were asked to check for each item whether their child *understands* or *speaks* the word — also when the child produces synonyms or pronunciation errors. The lists were fully digitised so caregivers could fill them out online. We scored the lists following the instructions of the manuals (Zink & Lejaegere, 2002, 2003). For the present study, we analyzed infants' word comprehension (i.e., the number of items for which caregivers checked *understands* or *speaks*) and total gestures (i.e., the total number of gestures for which caregivers checked *yes*, *sometimes*, or *often*). We did not take the different frequencies of these gestures into account but collapsed all *yes*, *sometimes*, and *often* responses, resulting in a number representing the sum of unique gestures in children's repertoires. In total, 98 caregivers filled out the N_YOUth_‐CDI 1 during Wave 1.

#### 2.2.3 Wave 2: N_YOUth_‐CDI 2

During Wave 2 when children were 2–4 years old, caregivers were asked to fill out a combined version of the short forms N‐CDI 2A (16–30 months) and N‐CDI 3 (30–37 months) (hereafter N_YOUth_‐CDI 2) (Zink & Lejaegere, 2003). The combined checklist resulted in a total number of 207 vocabulary items after removing the overlapping ones. The YOUth cohort study made the combined checklist because the second measurement wave covers a broad age range of children. Caregivers were asked to check the items that the child *speaks* — also in case the child produces synonyms or pronunciation errors. We replaced or removed 26 typical Flemish words with similar words that are more common in Standard Dutch spoken in the Netherlands (e.g., *bank* instead of *zetel/sofa* (“couch”)). We analyzed children's word production (i.e., the total number of items for which caregivers checked *speaks*). In total, 87 participants filled out the N_YOUth_‐CDI 2 during Wave 2.

#### 2.2.4 Wave 2: PPVT‐III‐NL

During the lab visit for Wave 2, we administered the third version of the Dutch Peabody Picture Vocabulary Test (PPVT‐III‐NL), which is a lab‐administered task of receptive vocabulary (Schlichting, 2005). The task measures whether a person can match a spoken word to one of the four pictures (i.e., multiple choice). It is designed as a behavioral task in which the participant points to one of the images and the experimenter produces the target words and scores manually. For the YOUth cohort study, we developed a computerised version of the PPVT‐III‐NL. The experimenter runs a script on a computer with a touch screen where children are provided with recordings of the test items and four pictures on the screen. This controls for differences in speaker pronunciations and minimises the role of the experimenter. Children can use the touch screen to select one of the pictures after the target item has been presented. During the task, words become increasingly more complex. The PPVT‐III‐NL has a total of 204 items, divided into 17 sets of 12 items. The task terminates when the child makes nine or more errors in one set (“final set”) (see Schlichting, 2005). The program automatically subtracts the number of errors from the maximum score (which is the number of the final set × 12 items), resulting in the child's raw score. During the test, the child's caregiver was present in the back of the room out of the child's view. Caregivers were explicitly instructed not to help or communicate with the child. We excluded five participants from the total sample regarding analyses involving the PPVT‐III‐NL, because these children did not cooperate during the task.

### 2.3 Coding

All coding of infants' behaviors and caregivers' responses was done in ELAN version 6.0 (Sloetjes & Wittenburg, 2008) for the multimodal corpus reported in van der Klis et al. (2023). A trained research assistant first annotated all infant vocalisations and gestures, using the coding scheme reported in van der Klis et al. (2023). Vocalisations were all sounds produced by the infants that were not vegetative or distress sounds. The gestures included in this study were (a) points (index‐finger extensions), (b) shows (holding out an object with extended arm(s) directed at the caregiver's face), and (c) gives (holding out an object with extended arm(s) directed at the caregiver's hands or in a way as to deliver the object to the caregiver). Whole‐hand points were excluded. We analyzed these deictic gestures because previous studies have identified that these gestures elicit many responses from caregivers and affect children's vocabulary outcomes (e.g., Choi et al., 2021; Donnellan et al., 2019; Wu & Gros‐Louis, 2015). For the present study, we collapsed the showing and giving gestures into one category (shows + gives) given the heterogeneity in the definitions of these gestures (following Cameron‐Faulkner et al., 2015). To assess inter‐annotator reliabilities, we report chance‐corrected modified Cohen's kappa (*κ*) using the built‐in calculator in ELAN which is based on the EasyDIAg toolbox (Holle & Rein, 2015). A random selection of 16 videos was double‐coded by the first author. In this subset, chance‐corrected kappa shows high agreement on infant vocalisations (*κ* = 0.92), infant points (*κ* = 1.0), and infant shows + gives (*κ* = 0.95).

After the offset of the infant gesture or vocalisation, a period of 2 seconds was analyzed for the caregiver response (following McGillion et al., 2013; Wu & Gros‐Louis, 2014). The onset of the response had to occur during or within this two‐second time frame. If not, the response was not considered temporally contingent and not included. In the case of a verbal response, we transcribed the utterance and annotated whether it was semantically contingent (i.e., a follow‐in response related to the infant's focus of attention) or not. We assumed the object or activity was in the infant's focus of attention when the infant was vocalising while either holding the object or playing with the object, performing the activity, looking at the object, and/or gesturing toward the object. For the segmentation and classification of caregivers' semantically contingent verbal responses, we found high agreement (*κ* = 0.88) between the research assistant and the first author. The verbal response was coded multimodal when the verbal response was coordinated with at least one nonverbal cue, including gestural, facial, or other bodily responses, following van der Klis et al. (2023). The nonverbal behavior had to overlap the verbal response at least partially in time. We found high agreement on the classification of multimodal responses (*κ* = 0.84). We coded “no response” when the infants' behavior did not elicit a verbal or nonverbal response from the caregiver within the two‐second period.

### 2.4 Analyses

**FIGURE 1:**
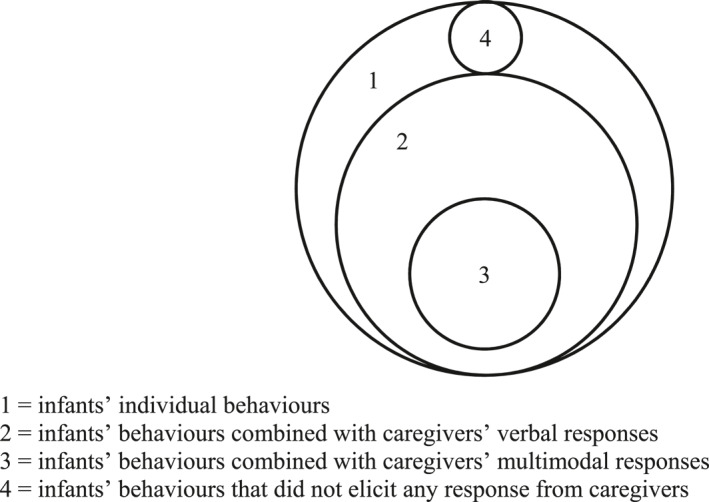
Four subsets of infant and dyadic behaviors used to predict children's vocabulary outcomes.

All analyses were carried out in *R* version 4.2.0 (R Core Team, 2022). We contrasted frequencies of four subsets of infant and dyadic predictors on children's concurrent and longitudinal vocabulary outcomes: (a) frequencies of infants' individual behaviors (vocalisations, points, and shows + gives) regardless of a caregiver response, (b) frequencies of infants' behaviors combined with caregivers' verbal responses, (c) frequencies of infants' behaviors combined with caregivers' multimodal responses, and (d) frequencies of infants' behaviors that did not elicit any response from caregivers. Infant behaviors included vocalisations, points, and shows + gives. Verbal responses only included semantically and temporally contingent verbal responses (following Donnellan et al., 2019). The third subset included only the verbally contingent responses that were coordinated with a nonverbal cue (e.g., gestural, facial, or bodily behavior) at least partially overlapping in time. For example, in subset 1, we analyze the frequency of infants' points. In subset 2, we only analyze the frequency of infants' points that elicited a verbal response from caregivers. In subset 3, we only analyze the frequency of infants' points that elicited a verbal response that is also combined with a nonverbal cue (i.e., multimodal response). Subset 4 includes infants' behaviors that did not elicit a response from caregivers. Figure 1 depicts these subsets of infant and dyadic behaviors. We examined which individual and/or dyadic behaviors predicted children's concurrent vocabulary comprehension and gesture repertoires (combined N‐CDI 1 & N‐CDI‐WG) at Wave 1 and vocabulary comprehension (PPVT‐III‐NL) and vocabulary production (combined N‐CDI 2A & N‐CDI 3) at Wave 2. We fitted robust generalised linear models using the package *robustbase* version 0.95–0 (Maechler et al., 2022). We used raw scores for all vocabulary outcomes. We added children's ages in weeks during Wave 1 (i.e., during the caregiver‐infant interaction task and the N_YOUth_‐CDI 1 measurement) and maternal education as a proxy for socio‐economic status (SES) rated on a 9–point scale as control variables to the models. This scale is based on the ISCED (International Standard Classification of Education) where 1 = no education and 9 = university degree, and all other levels of education in the Dutch system are represented in between. In the longitudinal models, we controlled for children's ages at both waves. By adding children's ages during Wave 1 and Wave 2 as predictors to the models, all other predictors are independent of the effects of children's age. All continuous predictors were centered and scaled.

Analysing whether frequencies of caregivers' responses predicted children's vocabularies while controlling for frequencies of infants' behaviors – as would be a necessary step in a mediation analysis – was difficult due to the high multicollinearity across predictors. Frequencies of infant behaviors (e.g., frequencies of infants' points) and frequencies of caregiver responses to these infant behaviors (e.g., frequencies of caregivers' responses to infants' points) are subsets of each other. Therefore, we compared the predictive value of individual behaviors versus dyadic behaviors in separate models instead. When we find that dyadic behaviors are stronger predictors of children's vocabularies compared to individual behaviors (by comparing the regression coefficients and statistical significance), this would suggest that dyadic behaviors are better predictors of children's vocabularies in this sample. We can also directly compare R‐squared values because the models have the same number of predictors fitted to the same dataset. Our approach thus uses the predictive value of infants' behaviors as a baseline against which to compare the predictive value of infants' behaviors combined with caregivers' verbal and multimodal responses to assess the relative contributions of dyadic versus individual behaviors.

## 3 RESULTS

### 3.1 Descriptive statistics

First, we present the frequencies of infant behaviors during the 6 minutes of free play at Wave 1 from the multimodal corpus reported in van der Klis et al. (2023). For the current study, we analyzed the concurrent and longitudinal vocabulary outcomes of these infants during Wave 2. The descriptive statistics of all measures can be found in Table 1. This shows that infants descriptively produced many more vocalisations than gestures during the 6 minutes of free play. All infants in the sample produced at least one vocalisation, but they did not all produce points or shows + gives. On average, children produced fewer than one of these deictic gestures per session. However, some children in the sample spontaneously produced four pointing gestures, while some children produced up to eight shows + gives. This suggests there is individual variability across children in their productions of vocalisations and gestures.

**TABLE 1:** Descriptive statistics of the frequencies of infants' points, shows + gives, and vocalisations during the caregiver‐infant interaction task and raw scores of vocabulary outcomes.

	*Wave*	*M*	*SD*	Range
Infant behaviors				
Points	1	0.22	0.68	0 – 4
Shows + Gives	1	0.61	1.57	0 – 8
Vocalisations	1	16.34	11.08	1 – 54
Vocabulary outcomes				
N_YOUth_‐CDI 1 comprehension	1	40.07	23.10	0 – 99
N_YOUth_‐CDI 1 gestures	1	19.18	7.87	6 – 49
N_YOUth_‐CDI 2 production	2	152.60	42.19	23 – 207
PPVT‐III‐NL comprehension	2	41.73	15.41	6 – 85

*Note*: The infant behaviors are derived from the multimodal corpus previously published in van der Klis et al. (2023).

Next, we examined caregivers' verbal and multimodal responses that were elicited by the infants' vocalisations and deictic gestures at Wave 1. The infants' behaviors were previously reported in the multimodal corpus in van der Klis et al. (2023). The descriptive statistics of caregivers' responses to the infants' behaviors are presented in Table 2. For the current study, we assessed how many infant behaviors elicited caregivers' semantically contingent verbal responses (2), the subset of caregivers' semantically contingent verbal responses that were coordinated with nonverbal behaviors (i.e., multimodal) (3), and the subset of infant behaviors that did not elicit any response (4).

**TABLE 2:** Descriptive statistics of subsets of caregivers' verbal and multimodal responses to infants' behaviors during the caregiver‐infant interaction task in Wave 1.

Infant behaviors	1. Total frequency *M* (*SD*)	2. Verbal responses *M* (*SD*)	3. Multimodal responses *M* (*SD*)	4. No responses *M* (*SD*)
Points	0.22 (0.68)	0.15 (0.48)	0.06 (0.24)	0.01 (0.09)
Shows + Gives	0.61 (1.57)	0.20 (0.69)	0.16 (0.62)	0.01 (0.13)
Vocalisations	16.34 (11.08)	5.22 (4.91)	2.55 (3.07)	2.22 (2.51)

More than two‐thirds of the total infant points elicited semantically contingent verbal responses from caregivers. For example, caregivers named a toy after their infant had pointed at it. This proportion is considerably higher than the proportion elicited by infants' shows + gives or infants' vocalisations. For the subset of caregivers' multimodal responses, the difference between infants' points and infants' shows + gives has become much smaller. Most caregiver responses elicited by infants' shows + gives are multimodal (18/23, or 78%). For example, caregivers name a toy while accepting it from the infant who gives them the toy. This is not the case for the contingent responses elicited by infants' points, where less than half were multimodal. Infants' vocalisations elicited a relatively smaller proportion of multimodal responses from caregivers compared to infants' gestures. Lastly, there was one (4%) point that did not elicit any response, there were two shows + gives (3%) that did not elicit any response, and there were 253 (14%) vocalisations that did not elicit any response from caregivers.

#### 3.1.1 Predicting vocabulary outcomes with infant behaviors regardless of responses

**FIGURE 2:**
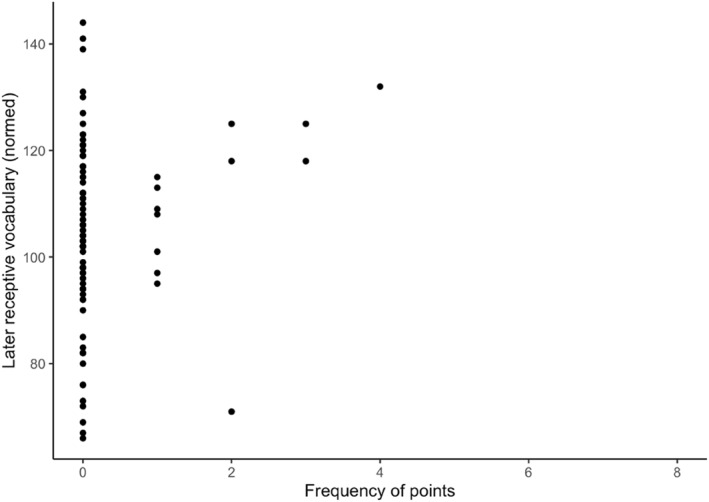
The relation between the frequency of infants' points and children's later receptive vocabularies.

First, we examined whether the frequencies of infants' behaviors (points, shows + gives, vocalisations) regardless of a caregiver response can predict children's concurrent and longitudinal vocabulary outcomes. The results are presented in Table 3. First, the results show that the frequency of infant behaviors regardless of a caregiver response cannot predict infants' concurrent gestures or their later productive vocabularies measured using N_YOUth_‐CDIs with children's age and maternal education controlled. For infants, we find significant negative effects of maternal education (*b* = −8.57, *SE* = 2.85, *p* < 0.01) and the frequency of infants' points on the N_YOUth_‐CDI 1 comprehension (*b* = −3.43, *SE* = 1.11, *p* < 0.01). Lastly, infants' points are positively related to children's PPVT‐III‐NL receptive vocabulary outcomes years later (*b* = 2.56, *SE* = 0.66, *p* < 0.001). This effect is depicted in Figure 2.

**TABLE 3:** Robust regression coefficients assessing influences of infant behaviors regardless of caregiver response on children's vocabulary outcomes with 95% confidence intervals.

	Wave 1		Wave 2	
	N_YOUth_‐CDI 1 comprehension	N_YOUth_‐CDI 1 gestures	N_YOUth_‐CDI 2 production	PPVT‐III‐NL comprehension
(Intercept)	39.35*** (34.89, 43.81)	18.29*** (17.09, 19.49)	156.98*** (145.62, 168.35)	41.66*** (39.47, 43.85)
Age at wave 1	9.25*** (5.10, 13.39)	4.09*** (3.00, 5.18)	4.99 (−4.32, 14.31)	−0.38 (−2.45, 1.70)
Age at wave 2	NA	NA	22.69*** (12.22, 33.17)	10.63*** (8.24, 13.03)
Maternal education	−8.57** (−14.23, −2.90)	0.17 (−0.99, 1.33)	−4.08 (−15.47, 7.32)	2.07 (−1.37, 5.50)
Points	−3.43** (−5.64, −1.22)	−0.21 (−0.96, 0.53)	−4.11 (−12.64, 4.41)	2.56*** (1.26, 3.87)
Shows + Gives	−1.37 (−4.60, 1.86)	0.72 (−0.68, 2.12)	1.54 (−7.35, 10.42)	1.02 (−0.89, 2.94)
Vocalisations	0.69 (−3.19, 4.57)	0.26 (−1.11, 1.64)	−0.10 (−6.27, 6.07)	0.85 (−0.81, 2.51)
Observations	96	96	86	104
Multiple *R* ^2^	0.22	0.37	0.38	0.52
Adjusted *R* ^2^	0.18	0.34	0.33	0.49
Residual Std. Error	21.95 (df = 91)	5.41 (df = 91)	27.76 (df = 80)	10.07 (df = 98)

*p < 0.05; **p < 0.01; ***p < 0.001.

### 3.2 Infant behaviors combined with caregivers' verbal responses

**FIGURE 3:**
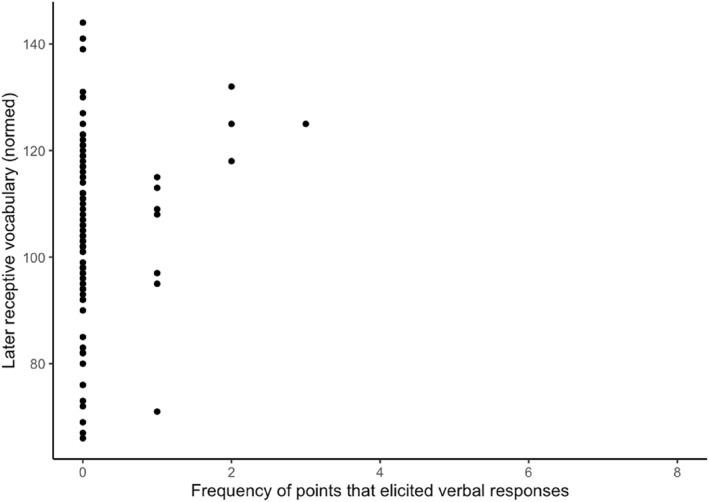
The relation between the frequency of infants' points that elicited verbal responses and children's later receptive vocabularies.

**FIGURE 4:**
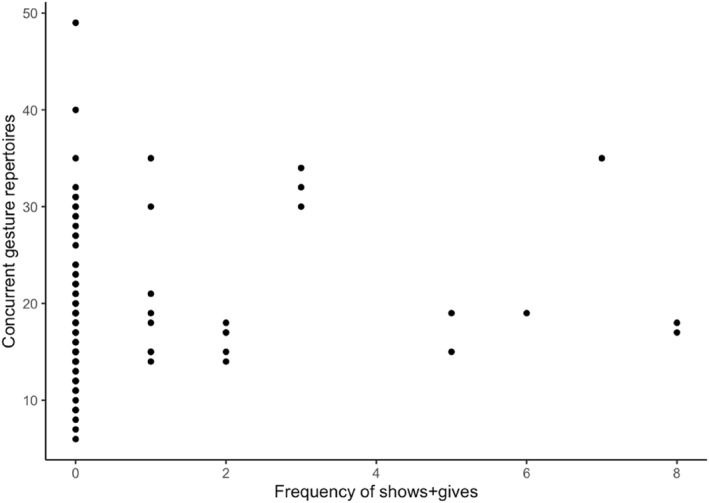
The relation between the frequency of infants' shows + gives and their concurrent gesture repertoires.

In the next set of models, we included the frequencies of infant behaviors that elicited a semantically contingent verbal response from caregivers. Apart from this, the reported models are identical to the previous models. The results are shown in Table 4. First, we found that infant points are still negatively related to the N_YOUth_‐CDI 1 comprehension (*b* = −3.63, *SE* = 1.60, *p* = 0.026) and positively related to children's later PPVT‐III‐NL receptive vocabularies (*b* = 2.42, *SE* = 0.56, *p* < 0.001) while controlling for children's ages and maternal education. Although comparable, Figures 2 and 3 show that the linear relationship between infants' points and children's later receptive vocabulary is less variable when examining infants' points that elicited verbal responses from caregivers. Although the regression coefficient of points that elicited verbal responses in Table 5 has decreased slightly compared to all points examined in Table 4, we do observe that the 95% confidence interval has become narrower when analysing points that elicited caregivers' verbal responses. We also found that, when taking infant behaviors that elicited semantically contingent verbal responses from caregivers into account, infants' shows + gives are positively related to their concurrent gesture repertoires (*b* = 1.53, *SE* = 0.35, *p* < 0.001), as shown in Figure 4. Infants' shows + gives regardless of a response do not predict infants' vocabulary skills as shown in Table 3, but higher frequencies of infants' shows + gives combined with caregivers' verbal responses have a positive effect on infants' concurrent gestures as shown in Table 4. The adjusted *R* ^2^ of the model predicting infants' concurrent gestures has also increased from 0.34 to 0.40.

**TABLE 4:** Robust regression coefficients assessing influences of the subset of behaviors that elicited verbal responses on children's vocabulary outcomes with 95% confidence intervals.

	Wave 1		Wave 2	
	N_YOUth_‐CDI 1 comprehension	N_YOUth_‐CDI 1 gestures	N_YOUth_‐CDI 2 production	PPVT‐III‐NL comprehension
(Intercept)	39.78*** (35.26, 44.30)	18.40*** (17.18, 19.62)	155.56*** (146.02, 168.04)	40.73*** (38.49, 42.98)
Age at wave 1	9.05*** (5.04, 13.06)	4.13*** (3.06, 5.21)	3.92 (−5.12, 12.95)	−0.21 (−2.31, 1.89)
Age at wave 2	NA	NA	23.53*** (12.07, 32.36)	10.96*** (8.37, 13.56)
Maternal education	−7.82** (−13.06, −2.59)	0.02 (−1.02, 1.07)	−2.10 (−11.69, 6.08)	1.89 (−1.90, 5.68)
Points	−3.63* (−6.83, −0.42)	−0.44 (−1.13, 0.25)	−2.11 (−14.10, 9.09)	2.42*** (1.30, 3.54)
Shows + Gives	1.01 (−4.44, 6.46)	1.53*** (0.83, 2.22)	6.16 (−1.70, 12.11)	0.50 (−1.23, 2.23)
Vocalisations	2.31 (−2.16, 6.79)	0.48 (−0.77, 1.74)	3.27 (−4.36, 10.25)	0.70 (−0.88, 2.29)
Observations	96	96	86	104
Multiple *R* ^2^	0.21	0.43	0.37	0.52
Adjusted *R* ^2^	0.17	0.40	0.32	0.49
Residual Std. Error	21.75 (df = 91)	5.22 (df = 91)	27.40 (df = 80)	9.78 (df = 98)

*Note:* *p < 0.05; **p < 0.01; ***p < 0.001.

**TABLE 5:** Robust regression coefficients assessing influences of the subset of behaviors that elicited multimodal responses on children's vocabulary outcomes with 95% confidence intervals.

	Wave 1		Wave 2	
	N_YOUth_‐CDI 1 comprehension	N_YOUth_‐CDI 1 gestures	N_YOUth_‐CDI 2 production	PPVT‐III‐NL comprehension
(Intercept)	39.65*** (35.10, 44.20)	18.41*** (17.18, 19.65)	156.11*** (146.82, 165.40)	40.80*** (38.46, 43.14)
Age at wave 1	8.65*** (4.65, 12.65)	4.17*** (3.12, 5.22)	4.70 (−3.29, 12.70)	0.04 (−2.26, 2.34)
Age at wave 2	NA	NA	21.58*** (12.74, 30.42)	11.03*** (8.41, 13.64)
Maternal education	−7.04** (−12.10, −1.98)	0.09 (−0.98, 1.15)	0.12 (−7.89, 8.13)	1.44 (−2.04, 4.91)
Points	−0.76 (−4.46, 2.95)	−0.31 (−1.11, 0.49)	−11.34 (−22.93, 0.25)	1.40 (−0.45, 3.24)
Shows + Gives	1.33 (−4.04, 6.69)	1.20** (0.44, 1.95)	4.19 (−2.15, 10.53)	0.61 (−0.83, 2.05)
Vocalisations	1.94 (−3.23, 7.11)	0.66 (−0.40, 1.72)	4.75 (−1.90, 11.40)	0.34 (−1.54, 2.21)
Observations	96	96	86	104
Multiple *R* ^2^	0.19	0.41	0.44	0.49
Adjusted *R* ^2^	0.15	0.38	0.40	0.46
Residual Std. Error	22.25 (df = 91)	5.41 (df = 91)	25.29 (df = 80)	10.79 (df = 98)

*p < 0.05; **p < 0.01; ***p < 0.001.

### 3.3 Infant behaviors combined with caregivers' multimodal responses

**FIGURE 5:**
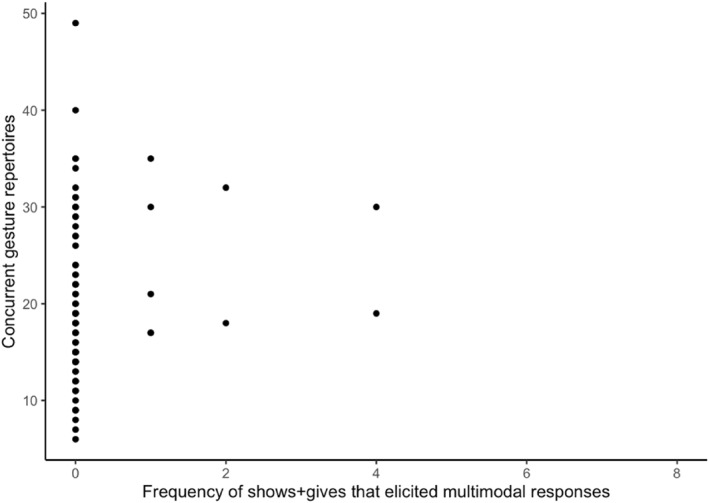
The relation between the frequency of infants' shows + gives that elicited multimodal responses and their concurrent gesture repertoires.

In the third set of models, we included the subset of infants' behaviors that elicited caregivers' contingent verbal responses coordinated with at least one nonverbal cue (i.e., a multimodal response). The results are reported in Table 5. First, we found that infants' shows + gives that elicited multimodal responses from caregivers positively related to children's concurrent gesture repertoires (*b* = 1.20, *SE* = 0.38, *p* < 0.01), as shown in Figure 5. Second, we found that when including only the number of infant points that elicited a contingent multimodal response from caregivers, this behavior does not significantly relate to children's N_YOUth_‐CDI 1 comprehension or later PPVT‐III‐NL outcomes anymore. Lastly, we observed that the model including infants' behaviors which elicited multimodal responses from caregivers fitted to children's N_YOUth_‐CDI 2 production has an increased adjusted *R* ^2^ compared to the previous two models, which indicates that these variables can explain more variance in the data.

### 3.4 Infant behaviors that did not elicit responses

In the last set of models, we included the subset of infants' behaviors that did not elicit any responses from caregivers. The results are reported in Table 6. We see reversed patterns from the previously reported models. First, we found a positive effect of points that did not elicit a response from caregivers on infants' concurrent word comprehension (*b* = 0.68, *SE* = 0.25, *p* < 0.01). We found a negative effect of shows + gives without a response on infants' concurrent gesture repertoires (*b* = −0.75, *SE* = 0.11, *p* < 0.001). Third, we found negative effects of points without a response on children's later expressive vocabulary (*b* = −5.81, *SE* = 0.73, *p* < 0.001) and receptive vocabulary (*b* = −0.40, *SE* = 0.16, *p* < 0.05). Lastly, we also found a negative effect of shows + gives without a response on children's later receptive vocabulary (*b* = −2.05, *SE* = 0.75, *p* < 0.01). The results are discussed below.

**TABLE 6:** Robust regression coefficients assessing influences of the subset of behaviors that did not elicit any responses on children's vocabulary outcomes with 95% confidence intervals.

	Wave 1		Wave 2	
	N_YOUth_‐CDI 1 comprehension	N_YOUth_‐CDI 1 gestures	N_YOUth_‐CDI 2 production	PPVT‐III‐NL comprehension
(Intercept)	39.55*** (34.96, 44.15)	18.46*** (17.15, 19.78)	157.36*** (146.28, 168.43)	40.94*** (38.70, 43.19)
Age at wave 1	8.46*** (4.33, 12.59)	3.98*** (2.89, 5.08)	4.49 (−3.89, 12.87)	0.07 (−2.19, 2.32)
Age at wave 2	NA	NA	21.35*** (10.61, 32.10)	11.22*** (8.57, 13.88)
Maternal education	−7.22** (−12.34, −2.10)	0.07 (−1.10, 1.23)	−3.78 (−11.68, 4.11)	0.82 (−2.12, 3.76)
Points	0.68** (0.19, 1.18)	0.00 (−0.11, 0.11)	−5.81*** (−7.26, −4.35)	−0.40* (−0.71, −0.09)
Shows + Gives	−0.22 (−1.19, 0.74)	−0.75*** (−0.98, −0.53)	−1.67 (−3.76, 0.41)	−2.05** (−3.55, −0.56)
Vocalisations	−0.03 (−4.11, 4.06)	0.46 (−1.18, 0.65)	−0.91 (−8.29, 6.47)	−0.33 (−2.00, 1.34)
Observations	96	96	86	104
Multiple *R* ^2^	0.18	0.37	0.39	0.50
Adjusted *R* ^2^	0.14	0.33	0.34	0.47
Residual Std. Error	22.28 (df = 91)	5.43 (df = 91)	28.52 (df = 80)	10.87 (df = 98)

*p < 0.05; **p < 0.01; ***p < 0.001.

## 4 DISCUSSION

The goal of this study was to assess whether infants' vocalisations and gestures combined with caregivers' verbal and multimodal responses are better predictors of children's vocabulary outcomes than infants' individual behaviors or infants' behaviors that did not elicit responses from caregivers. To examine this, we contrasted models with four different subsets of predictors on children's concurrent and longitudinal vocabulary outcomes: (a) frequencies of infants' individual behaviors (vocalisations, points, and shows + gives) regardless of a caregiver response, (b) frequencies of infants' behaviors combined with caregivers' verbal responses, (c) frequencies of infants' behaviors combined with caregivers' multimodal responses, and (d) frequencies of infants' behaviors that did not elicit any response from caregivers. This allowed us to pit two different theories that aim to explain how infants' behaviors relate to their language outcomes against each other. On the one hand, if the relationship between infants' gestures and spoken language is driven by their common underlying understanding of referentiality (e.g., Werner & Kaplan, 1963) or through an independent exploration of their vocal and gestural abilities (e.g., Long et al., 2022), we would expect infants' behaviors to predict children's language outcomes regardless of caregivers' responses. On the other hand, if infants' behaviors relate to their language development because they elicit informative responses from caregivers (e.g., Donnellan et al., 2019), we would expect dyadic behaviors to more robustly predict children's language outcomes. The results of this study improve our understanding of the facilitative role of infants' prelinguistic vocalisations and gestures in their language development.

### 4.1 Infant points predict long‐term vocabulary outcomes

First, when examining infants' behaviors regardless of caregivers' responses, we found that only infants' points are positively related to children's receptive vocabulary skills measured several years later. The finding that infants' points are a robust predictor of children's longitudinal vocabulary outcomes agrees with previous studies, as infants' points have often been found to predict children's concurrent and longitudinal vocabulary outcomes (for meta‐analyses, see Colonnesi et al., 2010; Kirk et al., 2022). In the current study, we did not find a concurrent relationship between the frequency of infants' points and children's gesture repertories. The cross‐linguistic mean age of acquisition for the pointing gesture is 10.4 months of age (Frank et al., 2021). Therefore, it is likely that the infants in our study who pointed have only started doing so recently. When infants' points facilitate language learning because they elicit responses from caregivers which provide infants with learning opportunities, we expect that it would take some time before the facilitative effect on children's vocabulary size can manifest. The meta‐analyses revealed that the relationship between infants' points and children's vocabulary becomes stronger with age (Colonnesi et al., 2010; Kirk et al., 2022). The results of our study suggest that, although infants' points measured around 9–11 months of age do not predict infants' concurrent gesture repertoires, they are predictive of children's long‐term receptive vocabulary outcomes.

The results additionally show that infants' points are negatively associated with infants' concurrent word comprehension skills. One possible explanation is that infants who produce more gestures do this to compensate for reduced vocabulary. This strategy is, for example, found for bilingual school‐aged children who tend to use more gestures than their monolingual peers (e.g., Nicoladis et al., 2009). It could be possible that such compensation strategies provide infants with long‐term benefits, causing them to eventually learn new words faster. This could further explain why these infants end up having larger vocabularies at 2–4 years. Yet, the compensation strategy cannot explain why infants who produce points without eliciting responses from their caregivers have larger concurrent receptive vocabularies as reported by their caregivers. As the latter finding is based on very few datapoints, a more in‐depth, longitudinal research of infants is needed to further explore these relationships.

When taking the instances of infants' points which elicited caregivers' verbal responses into account, the variable remains a significant predictor of children's later receptive vocabularies. More than two thirds of infants' points elicited contingent responses from caregivers, so these predictors (infant points regardless of a caregiver response and infant points that elicited verbal responses from caregivers) are rather similar. Yet, the fact that losing over 30 per cent of data points did not impact the predictive value of points suggests that either 1) infants' points are a very robust predictor of children's long‐term receptive vocabularies and/or 2) the relationship between infants' points and their vocabulary outcomes is driven by the contingent verbal responses that infants' points tend to elicit from caregivers. In support of the latter hypothesis, previous studies found that infants' points tend to elicit more labeling responses from caregivers (Kishimoto et al., 2007; Wu & Gros‐Louis, 2015). There is a frequent pattern of an infant pointing at a toy and the caregiver immediately naming that toy after the infant expressed interest in it. It could be possible that hearing frequent object labeling, particularly for objects that infants are interested in, improves children's word comprehension skills. When infants produce many pointing gestures, they create many word‐learning opportunities for themselves. Although the models fitted with infants' behaviors that did not elicit any caregiver responses should be interpreted with caution due to the low number of data points, the initial findings show that infants' points without responses are negatively associated with children's long‐term vocabulary outcomes. These patterns are in favor of the hypothesis that caregivers' contingent feedback on infants' gestures are a key component of the positive associations between early gestures and language development.

Yet, it is difficult to tease apart the effects of points from the effects of infants' points combined with caregivers' contingent responses in naturalistic caregiver‐infant interactions, because infants' points tend to elicit high rates of contingent verbal responses, making the two variables rather similar. Therefore, these results cannot completely rule out that the relationship between points and later vocabulary reflects the common underlying understanding of symbolic and referential use of gestures and words (see Werner & Kaplan, 1963). Nevertheless, the high prevalence of caregivers' contingent verbal responses to infants' points, the finding that removing one‐third of points which did not elicit responses from caregivers did not negatively impact their predictive value for children's vocabulary outcomes, coupled with the finding that those points that did not elicit any caregiver responses are negatively related to children's long‐term vocabulary outcomes, together strongly suggest that infants' points facilitate children's language development through their tendency to elicit verbal responses from caregivers at opportune moments.

### 4.2 Dyadic shows + gives predict children's gesture repertoires

Recently, Choi et al. (2021) found that for 10‐month‐olds, shows + gives is a better predictor of children's later vocabulary skills than points, but shows + gives was not a significant predictor anymore by 12 months. Only from 14 months onwards, infants' points became a significant predictor. In contrast to Choi et al. (2021), we did not find significant effects of infants' shows + gives on children's concurrent or longitudinal vocabulary skills when including all behaviors regardless of caregivers' responses. The children included in the present study are of a broader age range (9–11 months), and gestures develop rapidly during this period (e.g., Frank et al., 2021). In addition, the children in our study are from predominantly high SES backgrounds while the children in Choi et al. (2021) were from diverse backgrounds. Infants from higher SES families generally start producing points earlier (Rowe & Goldin‐Meadow, 2009) which could speed up their vocabulary development. The high SES infants in our study could show faster progression in their gesture development; and therefore, points could have already gained more predictive value than shows + gives. Nevertheless, the results of our study do not show that 9‐ to 11‐month‐old infants' shows + gives significantly correlate with any of the concurrent or longitudinal vocabulary measures.

We also examined whether infants' shows + gives that elicited contingent responses from caregivers are better predictors of children's vocabulary outcomes compared to infants' shows + gives separately. We found that only infants' shows + gives combined with caregivers' verbal and multimodal responses are significant predictors of children's concurrent gesture repertoires. Infants' gesture repertoires positively influence children's longitudinal vocabulary skills (e.g., Fenson et al., 2007), but the influence of infants' shows + gives combined with caregivers' contingent responses on children's gesture repertoires at 9–11 months may not have been large enough to also show a facilitative effect on children's longitudinal vocabulary outcomes in the present study. Nevertheless, we still observed an increase in the variation explained in the model predicting children's expressive vocabulary outcomes when fitting infants' behaviors which elicited multimodal responses from caregivers. Infants' gives tend to elicit higher proportions of multimodal responses from caregivers compared to other gestures (van der Klis et al., 2023). For example, the caregiver accepts the object while talking about it. The combination of talking about an object while touching the object could boost children's word‐learning abilities. Recently, Chen et al. (2021) showed that touching of a named object by the caregiver increases the duration of children's looks at the toy. By talking about the object and interacting with it physically, this type of response is likely to establish joint attention. Such responses also satisfy the infant's desire to share attention and interest with others (Boundy et al., 2019). In addition, naming combined with a nonverbal cue toward or with the object could reduce the referential ambiguity in the learning context (Baldwin et al., 1996; Grassmann & Tomasello, 2010; Kory Westlund et al., 2017; Verhagen et al., 2019). Since infants' shows + gives tend to elicit high rates of multimodal responses from caregivers, they could facilitate children's vocabulary outcomes by reducing referential ambiguity in unclear or novel speech, by clearly establishing joint attention between the infant and the caregiver, and/or by enhancing the infants' attention to the toy.

Lastly, we found that infants' shows + gives that did not elicit any responses from caregivers are negatively associated with infants' gesture repertoires and their long‐term receptive vocabulary outcomes. Again, this finding carefully points us in the direction that infants' gestures are beneficial to language development only when caregivers provide contingent responses to them. When caregivers fail to respond contingently to infants' gestures, this results in their infants using fewer types of gestures concurrently and knowing the meanings of fewer words in the long run.

### 4.3 Infant vocalisations do not predict vocabulary

We did not find any effects of infants' prelinguistic vocalisations on children's vocabulary outcomes. In a previous study, 11‐month‐old infants' gaze‐coordinated and responded‐to vocalisations were the best predictors of children's vocabularies (Donnellan et al., 2019). Less than a quarter of all infant vocalisations were gaze‐coordinated in the study by Donnellan et al. (2019). In our study, individual infants' vocalisations regardless of a caregiver response versus infants' vocalisations combined with a caregiver response did not influence children's vocabulary outcomes either way. It could be possible that we did not find an effect because we did not measure infants' gaze direction during vocalising. While gaze‐coordination results in higher response rates from caregivers (Donnellan et al., 2019), measuring infants' vocalisations combined with caregivers' responses regardless of gaze did not have any predictive value for children's vocabulary skills in our study. It could be possible that measuring gaze to determine children's communicative intent is less important for infant gestures than infant vocalisations. Infants produce far more vocalisations than gestures, and it may be more difficult for caregivers to determine when children produce vocalisations with the intention to communicate or to determine their communicative goal. Another explanation is that there are fast developmental changes across children within the age range (9–11 months). The children in the study by Donnellan et al. (2019) were slightly older and produced more advanced types of Consonant‐Vowel (CV) vocalisations (i.e., canonical babbles) compared to non‐CV vocalisations. Although Donnellan et al. (2019) did not find differences between CV and non‐CV vocalisations, the grouped variable including all types of vocalisations used in the present study could gain more predictive value when this variable includes more canonical babbles at an older age. The predictive value of infants' vocalisations and gestures could therefore change continuously across children's development.

### 4.4 Multimodality does not further improve predictability

Contrary to our expectations, we found that the subset of infants' behaviors that elicited multimodal responses does not further improve the predictive value compared to infants' behaviors that elicited verbal responses. First, when examining infants' points that elicited multimodal responses, these behaviors cannot predict children's receptive vocabulary outcomes anymore. This suggests that the predictive value of infants' points does not stem from the multimodal nature that caregivers' responses may have. Rather, we found evidence that infants' points are related to children's receptive vocabularies regardless of caregivers' responses. Yet, infants' points elicit such high rates of contingent verbal responses from caregivers in this naturalistic dataset, that it is difficult to tease apart the effects of infants' points from the effects of points that elicited caregivers' verbal responses. Second, we found that infants' shows + gives which elicited verbal or multimodal responses from caregivers positively relate to infants' gesture repertoires. This tentatively suggests that the facilitative effect of caregivers' responses for children's language development may depend on the type of infant behavior. While infants' points may be beneficial for children's receptive vocabulary through their tendency to elicit verbal responses from caregivers – which are often labeling responses – infants' shows + gives may be beneficial through their general tendency to elicit responses that can be both verbal or multimodal in nature. Importantly, the lack of significant results could also reflect a loss of statistical power. Only approximately a quarter of infants' points elicited multimodal responses from caregivers. It could be possible that we did not find the expected effect because we do not have enough data of behaviors that elicited multimodal responses from caregivers. Therefore, the additional value of nonverbal information in caregivers' responses for predicting children's vocabulary outcomes warrants replication in a larger dataset.

### 4.5 Negative effect of maternal education

We found that maternal education is negatively related to infants' concurrent word comprehension skills. This result is in contrast with many previous studies that show that maternal education is positively associated with children's vocabulary size (e.g., Feldman et al., 2000; Fenson et al., 2007; Hoff, 2003). However, there are also previous studies reporting a negative effect of maternal education on caregiver‐reported word comprehension (e.g., Feldman et al., 2000; Reese & Read, 2000). This effect is likely caused by a caregiver reporting bias on the CDIs, where lower SES caregivers may overreport their infants' vocabularies because they think larger vocabularies are more desirable or higher SES caregivers may underreport their children's vocabularies because they underestimate their infants' language skills, resulting in a negative SES effect. The negative effect of maternal education on caregiver‐reported word comprehension is typically only found for infants. Reports for infants may be more susceptible to biases because infants' language use and understanding requires more interpretation by the caregiver.

### 4.6 Limitations and future directions

The current study has several limitations that should be addressed in future studies. Even though the sample in our study is large, it overrepresents highly educated, white, western caregivers. Caregivers' education is known to affect infants' gestures, caregivers' contingent speech, and children's vocabulary outcomes (e.g., Hoff, 2003; McGillion et al., 2017; Rowe & Goldin‐Meadow, 2009). Therefore, it is important to verify our findings in a more socio‐economically diverse sample, especially concerning the finding that maternal education and the frequency of infants' points are negatively associated with infants' vocabulary comprehension. In addition, caregivers from different cultures may not all be equally talkative to their infants (see Cristia et al., 2019) which could also influence their response rates to infants' prelinguistic behaviors. Future studies should determine whether infants' points and infants' shows + gives still relate to children's vocabulary outcomes in diverse cultures where adults are less responsive to infants. If infants' gestures still relate to their vocabulary outcomes in these cultures, this suggests that mechanisms other than learning from caregivers' responses are also at play.

In addition, we only examined one setting (i.e., free play) at one point in time (i.e., at 9–11 months of age) in the current study. Infants and caregivers are both likely to change their behaviors depending on the environment. For example, we would expect infants to use more referential gestures when objects are further away or out of reach. During book reading, infants could be more likely to use points to refer to different pictures on the pages, while infants' shows + gives were most common during free play with a set of toys. Subsequently, caregivers' responsiveness will be influenced by differences in infants' behaviors (van der Klis et al., 2023). In addition, we may expect the influence of different dyadic combinations of behaviors on children's vocabulary outcomes to change across children's development. As shown by Choi et al. (2021), the predictive value of infants' points and infants' shows + gives for their vocabulary outcomes changes from 10 to 14 months. Caregivers' responsiveness also changes over time. For example, Choi et al. (2021) showed that caregivers respond more often to 10‐month‐old shows + gives than points. However, by 14 months, caregivers respond to both deictic gestures equally often. In sum, dyadic behaviors are expected to change across different settings and children's developmental stages – differentially predicting children's vocabulary outcomes.

We also want to draw attention to the large age range of children included in Wave 2. This was a design choice made by the YOUth cohort study. From previous studies, we have learned that the predictive value of early infant behaviors for later vocabulary outcomes can change over time (e.g., Choi et al., 2021). In addition, predictive relations between, for example, infants' pointing gestures and children's vocabulary outcomes increase with age (e.g., Colonnesi et al., 2010). By statistically controlling for children's ages during Wave 1 and Wave 2 in our longitudinal models, we ensured that none of the longitudinal effects are driven by children's ages during testing. However, this does not guarantee that the long time interval between Wave 1 and Wave 2 for parts of the sample may have obscured some of the longitudinal relations in the data.

Lastly, it is important to note that the patterns which emerged in the results are based on limited data. Many infants in our study did not produce any points or shows + gives during the play session. Importantly, this did not inform us much about their predicted vocabulary size. We only find that the infants who *do* produce these gestures naturally at such an early age, when their caregivers respond contingently, are more likely to have larger vocabularies later. The young age of the infants (9–11 months) and the setting (a semi‐naturalistic play sessions without the intent to elicit gestures) resulted in a small number of annotated gestures. This also made it more difficult to tease apart all the different effects: the behaviors themselves, infant behaviors which elicited verbal responses, infant behaviors which elicited multimodal responses, and infant behaviors which did not elicit any response. Due to the low frequency, we could not reliably compare the predictive value of infant behaviors that elicited nonverbal responses to the models presented in this study. Therefore, we cannot claim that multimodal responses have an added value that goes beyond the added value of nonverbal responses. Caregivers' do not often give nonverbal responses without a verbal component (see van der Klis et al., 2023). We would need a much larger annotated dataset to tease apart any additional effects of multimodal behaviors from the addition of nonverbal behaviors by themselves. This is an important question that needs to be addressed in future studies.

A significant contribution of our study is that we have provided evidence for the predictive value of infants' deictic gestures, including points and shows + gives, measured at a young age (9–11 months of age) on children's long‐term vocabulary outcomes (2–4 years of age). Although measured early, the facilitating effects of infants' gestures on their vocabulary development remain present all through the crucial years of rapid vocabulary development. Then, we showed that the predictive value of some infant behaviors can increase depending on whether they elicited contingent responses from caregivers during free play. The results suggest that infants' points tend to elicit verbal responses from caregivers which facilitate children's word comprehension skills, while infants' shows + gives tend to elicit verbal or multimodal responses from caregivers which positively correlates with infants' concurrent gesture repertoires. These results suggest that specific dyadic combinations of infants' gestures and caregivers' verbal and multimodal responses are more robust predictors of children's long‐term vocabulary outcomes than infants' behaviors alone. We also provide some initial evidence that infants' gestures that did not elicit any responses from caregivers are negatively associated with children's long‐term vocabulary outcomes, although this should be replicated in a larger dataset. Although the evidence is by no means conclusive, the data in this study provide new insights supporting the hypothesis that infants' gestures are related to their language development through contingent feedback from caregivers.

## AUTHOR CONTRIBUTIONS

**Anika van der Klis**: Conceptualization; data curation; formal analysis; methodology; writing ‐ original draft. **Caroline Junge**: Conceptualization; supervision; writing ‐ review & editing. **Frans Adriaans**: Conceptualization; methodology; supervision; writing ‐ review & editing. **René Kager**: Conceptualization; funding acquisition; methodology; supervision; writing ‐ review & editing.

## CONFLICT OF INTEREST STATEMENT

The authors declare no conflicts of interest with regard to the funding source for this study.

## Data Availability

YOUth is a longitudinal study that aims to produce and safely store FAIR and high‐quality data. The data can be accessed for both use and verification purposes upon request (see https://www.uu.nl/en/research/youth‐cohort‐study/data‐access). The preregistration, *R* script, and other materials can be found online: https://osf.io/zxnqd/.
